# Smoking and nicotine exposure delay development of collagen-induced arthritis in mice

**DOI:** 10.1186/ar2728

**Published:** 2009-06-11

**Authors:** Sofia S Lindblad, Piotr Mydel, Ing-Marie Jonsson, Robert M Senior, Andrej Tarkowski, Maria Bokarewa

**Affiliations:** 1 Department of Rheumatology and Inflammation Research, University of Gothenburg, Sahlgrenska University Hospital, Guldhedsgatan 10, Göteborg, S-41346, Sweden; 2 Department of Medicine, Division of Pulmonary and Critical Care Medicine, Washington University, 660 S. Euclid Avenue, Campus Box 8052 St. Louis, Missouri 63110, USA

## Abstract

**Introduction:**

Recent epidemiologic studies have implicated smoking as an environmental risk factor for the development of rheumatoid arthritis (RA). The aim of the present study is the evaluation of the role of cigarette smoke (CS) in the pathogenesis of collagen-induced arthritis in mice.

**Methods:**

DBA/1 mice exposed to CS for 16 weeks (n = 25) and mice exposed to nicotine in drinking water (n = 10) were immunized with collagen type II (CII). Severity of arthritis was evaluated clinically and morphologically and compared with control mice (n = 35). Intensity of inflammation was evaluated by serum IL-6 and TNF-α levels. Additionally, antibody response to CII (anti-CII) and citrullinated peptides (aCCP) was measured.

**Results:**

Clinical evaluation of arthritis showed a delayed onset of arthritis in CS-exposed mice compared with non-smoking controls (*P *< 0.05). Histologic index and weight changes were comparable between the groups; however, smoking mice presented less weight loss during the acute phase of the disease and gained weight significantly faster in the recovery phase (*P *< 0.05). Similar results were obtained in the mice exposed to nicotine. Nicotine also showed a direct anti-inflammatory effect diminishing IL-6 production by stimulated splenocytes *in vitro *(*P *< 0.001). Additionally, smoking mice had lower levels of aCCP and anti-CII antibodies compared with non-smoking (*P *< 0.05).

**Conclusions:**

Neither smoking nor nicotine exposure aggravates development of CII-induced arthritis in mouse model. Moreover, CS exposure was associated with a lower level of anti-CII antibodies, providing a possible explanation for a delay of arthritis onset in this group.

## Introduction

Rheumatoid arthritis (RA) is an autoimmune disease characterized by severe joint inflammation, joint destruction, and disability. The disease develops as a result of a multilayer interplay between environmental and genetic factors. These processes are mediated by both innate and acquired immune systems [1]. Cigarette smoking is considered a risk factor in the development of RA [2] and an influence on joint damage [3]. An excessive citrullination of peptides in the lungs of smokers has been suggested as a direct link between smoking and the formation of antibodies to citrullinated peptides, which can be found in approximately 60% of RA patients [4-6] and were also shown to contribute to the development of experimental arthritis [7,8]. Conversely, smoking has also been shown to have a beneficial effect on several autoimmune disorders. In a retrospective study of the autoimmune skin disease pemphigus, smokers went into partial or complete remission significantly more often than non-smokers [9]. Analysis of patients with primary Sjögren's syndrome showed that smokers had a lower degree of focal inflammation in minor salivary glands [10]. Smoking was also shown to be protective to cartilage degeneration in osteoarthritis [11], probably due to the stimulating effect of nicotine on collagen and glycosaminoglycan synthesis in chondrocytes [12].

One of the key substances in cigarette smoke, nicotine, has been shown to have potent anti-inflammatory effects in experimental ulcerative colitis [13], to improve outcome of sepsis through its effect on inflammatory mediators [14], to block leukocyte recruitment [15], and significantly inhibit lipopolysaccharide (LPS)-induced TNFα and IL-6 production as well as splenocyte proliferation [16]. Many of these effects are assigned to the activation of the cholinergic autonomous nervous system [14,15]. Nicotine dependence has been shown to be mediated by, among others, the glutamergic system and preclinical data shows that substances lowering glutamergic neurotransmission decrease nicotine self-administration in laboratory animals [17]. It is worth mentioning that levels of glutamate in synovial fluid are significantly higher in patients with active arthritis [18], as well as in synovial tissue from rat with collagen-induced arthritis (CIA) [19].

A lack of published experimental studies concerning the relevance of smoking to the pathophysiology of RA encouraged us to determine if prolonged exposure to cigarette smoke in controlled conditions would affect the incidence and disease severity of CIA in mice. In our study we used a well-established model of smoke-induced emphysema in mice [20,21]. In the course of this model, mice develop a progressive infiltration of inflammatory cells including dendritic cells, neutrophils, macrophages, and lymphocytes into the lung tissue followed by destruction of alveolar spaces. These processes have major similarities to the histologic changes described in resected lung tissues of smokers [22]. Accordingly, we exposed adult DBA/1 mice to smoke from four non-filtered cigarettes a day for 16 weeks, according to an established animal model of smoking-induced emphysema [20], then immunized them with collagen II-inducing CIA and continued the smoking regimen until the end of the experiment. For the nicotine experiment, adult DBA/1 mice with CIA were employed and supplemented with nicotine in water from day one of first immunization and throughout the course of the experiment. Due to the fact that triggering CIA is fully T-cell dependent [23] and T-cell null mice are resistant to CIA, we also tested the response of mice splenocytes to various inflammatory substances in the presence and absence of nicotine.

We demonstrate with this study that smoking not should be considered a direct risk factor for the development of autoimmune arthritis as it delays onset and even slows down the progression of destructive arthritis.

## Materials and methods

### Mice

Male DBA/1 mice (age 6 to 8 weeks, n = 70; Taconic USA, Taconic Europe A/S, Ry, Denmark) were used in the CIA experiments. For *ex vivo *studies on the effect of nicotine on splenocytes, NMRI male mice (B&K Universal, Sollentuna, Sweden) were used. All animals were kept under standard environmental conditions and had free access to standard laboratory food and drinking water. Ethical permission was obtained from the Animal Research Ethics Committee of Göteborg University and Animal Studies Committee of Washington University in St. Louis, MO, USA.

### Collagen-induced arthritis

Chicken collagen II (Sigma St. Louis, MO, USA) was dissolved to a concentration of 2 mg/ml in 0.1 M acetic acid. DBA/1 mice were immunized at the base of the tail by subcutaneous injection with 100 μg collagen II emulsified in an equal volume of complete Freund's adjuvant (Sigma St. Louis, MO, USA). Booster immunization with 100 μg of collagen II in incomplete Freund's adjuvant was administered on day 21 after first immunization. Mice were regularly weighed and checked for the development of arthritis. Clinical evaluation of joints for the signs of arthritis was performed daily. Animals (smoking n = 25, nicotine n = 10, and control n = 35) were sacrificed at day 42 to 46 following first immunization. Blood samples were taken from v jugularis for serologic analyses of cytokines, anti-collagen II antibodies and anti-cyclic citrullinated peptides (aCCP). Paws were taken for histologic evaluation and assessed for synovitis and erosion in joints.

### Cigarette smoke exposure

DBA/1 mice (n = 25) were subjected to the smoke from four unfiltered University of Kentucky 2R1 Research cigarettes per day), six days per week for 16 weeks prior to immunization, with the use of a smoking apparatus according to an established model of smoking-induced emphysema [20,21]. The amount of nicotine provided to the mice (850 μg/cigarette, 4 cigarettes/day) correspond to a smoking regimen of more than one pack per day in the human setting. The smoking regimen was continued throughout the course of the experiment (for six more weeks) giving a total of 22 weeks of cigarette smoke exposure.

### Nicotine supplementation

To study the influence of nicotine on the development of CIA, DBA/1 mice (n = 10) were subjected to supplementation of nicotine in drinking water. Nicotine as a free base (Sigma St. Louis, MO, USA) was added to tap water, 100 μg/ml (concentration 0.01%), and given as the only available fluid to mice from the day of immunization and throughout the experiment.

### Clinical evaluation of arthritis

To assess the intensity of arthritis, a clinical scoring system of 0 to 3 points for each paw was used [24]: 0 = no sign of inflammation; 1 = mild swelling and/or erythema; 2 = moderate swelling and erythema; 3 = marked swelling and erythema. The arthritic index was constructed by adding the scores from all four limbs for each animal. The frequency of arthritis indicates a proportion of mice exhibiting any signs of clinical arthritis.

### Histologic examination

Tissue section of all four paws from the DBA/1 mice that had been excised at the end of the experiment were imbedded in paraffin cut in 3 μm thick slices and stained with H&E. The sections were evaluated by a blinded examiner (SSL, I-MJ, MB) for synovitis and erosion of bone/cartilage. Synovial hypertrophy (synovitis) was defined as a membrane thickness of more than two cell layers. A histologic scoring system of synovitis was used as follows: 1 = mild; 2 = moderate; and 3 = severe [25]. Destruction of cartilage and subchondral bone was registered separately. Knee joints, ankles, elbows, and wrists were inspected, and a mean score from all inspected paws per animal was calculated.

### Impact of nicotine on *in vitro *cell responses

Naive NRMI mice splenocytes were isolated and incubated in complete medium (Iscove's modified Dulbecco's medium enriched with 50 μg/ml gentamycin (Sigma St. Louis, MO, USA), 4 mM L-glutamine (Sigma St. Louis, MO, USA), 50 μM mercaptoethanol (Sigma St. Louis, MO, USA), and 10% FCS (Biological Industries, Beit Haemek, Israel) until use. Cells were seeded onto 96-well plates (1 × 10^6^/ml) in 100 μL of Iscove's modified Dulbecco's medium (Sigma St. Louis, MO, USA) and incubated with nicotine at 0 to 100 μg/ml and LPS (Sigma St. Louis, MO, USA) at 1 to 100 μg/ml. After 24 hours of incubation, the supernatants were collected and frozen at -70°C for future analysis.

### Measurements of antibody and cytokine levels

Quantification of anti-collagen II antibodies in serum was performed as described elsewhere [25]. Levels of aCCP in sera were measured using Immunoscan CCPlus^® ^(Euro-Diagnostica, Malmö, Sweden) with modifications. Mouse sera diluted 1/25 was applied to the wells coated with a mixture of citrullinated synthetic peptides provided by the manufacturer. Peroxidase-conjugated rabbit anti-mouse IgG specific for gamma chain antibodies was used for detection (Dako A/S, Glostrup, Denmark). Values of antibodies are expressed in relative units with 100 corresponding to negative result (< 25 U). IL-6 levels in supernatants from stimulated splenocytes were measured by a bioassay employing IL-6 sensitive B9 cells as described elsewhere [25] and TNFα was quantified using a standard ELISA assay (R&D Systems, Minneapolis, MN, USA).

### Statistical analysis

Statistical evaluation was made using the Mann–Whitney U test, the chi-squared test or Student's t test. Values are reported as medians and interquartile ranges or means ± standard error of the mean.

## Results

### Cigarette smoke exposure delays the onset and progression of collagen-induced arthritis

**Figure 1:**
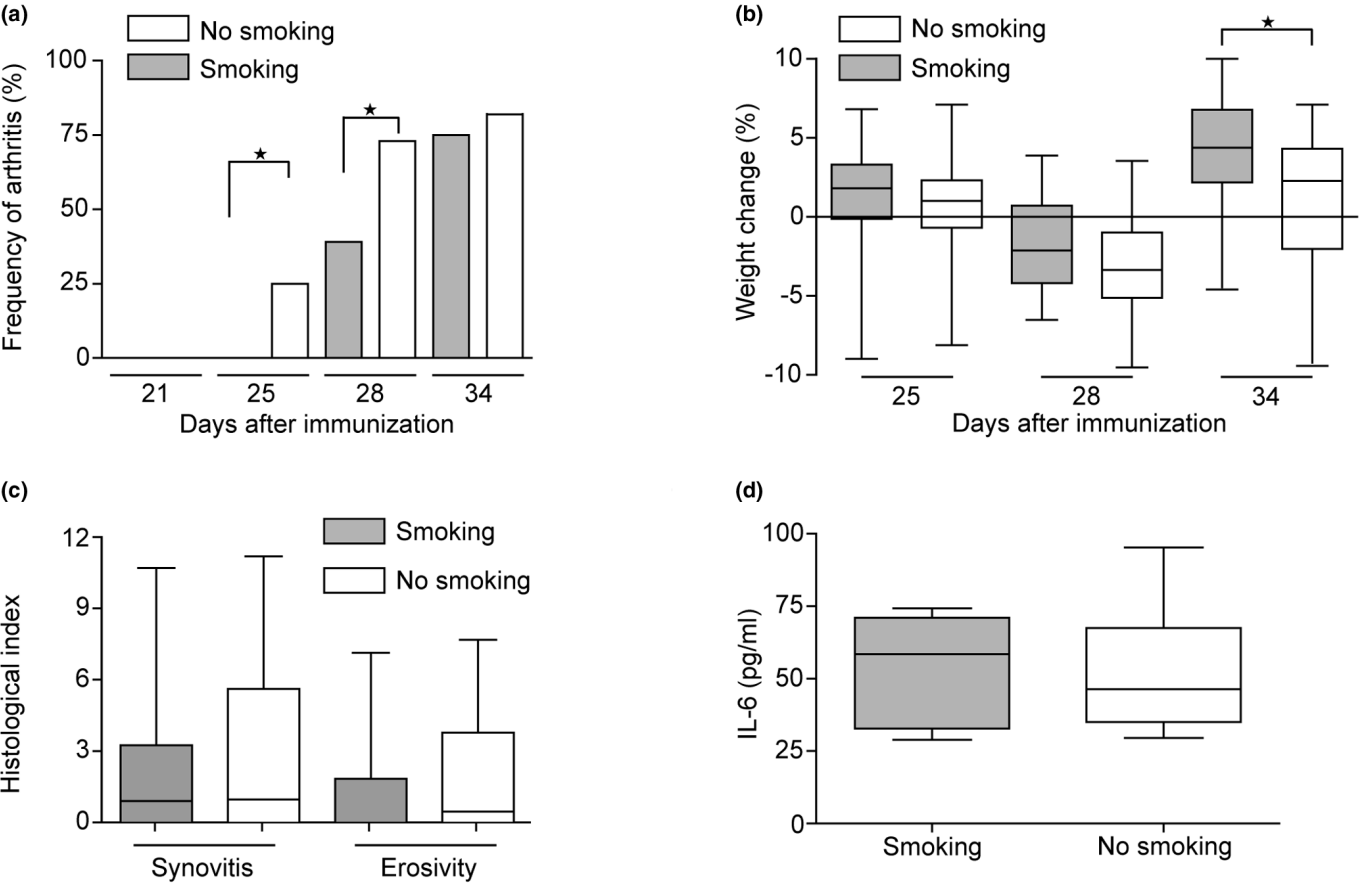
Delayed development and progression of collagen-induced arthritis in DBA/1 mice subjected to prolonged cigarette smoke exposure. **(a) **Clinical signs of arthritis were followed between days 21 and 46 after immunization. Statistical evaluation was performed using chi-squared test. Development of arthritis was significantly delayed in smoking animals (*P *< 0.05). **(b) **Weight development after immunization with collagen II. Weight change was calculated as the change in percent as compared with the day of booster injection with collagen II. **(c) **Histologic changes in the joints were evaluated at day 46. **(d) **Levels of IL-6 were measured in serum at day 46 after first immunization. **(b to d) **Statistical evaluation was made using Mann-Whitney U test. Values are presented as mean ± standard error of the mean. Horizontal lines indicate medians. Data from two independent experiments were pooled (n = 50).

Two independent experiments were performed containing 20 and 30 mice, respectively. Daily clinical evaluation of joints was performed in the course of the experiments. The onset of CIA and its development pattern was identical in both experiments, so the results of these two experiments were pooled and are presented in Figure 1. The results revealed that mice exposed to cigarette smoke developed arthritis significantly later and to a less extent than non-smoking mice. At day 25 after immunization, 25% of the controls showed arthritis whereas none of the mice exposed to cigarette smoke had signs of joint inflammation (*P *< 0.05). This significant difference persisted to day 28 with nearly 75% of control animals affected as compared with less than 30% for the mice exposed to cigarette smoke (*P *< 0.05; Figure 1a). However, by day 34 the mice exposed to cigarette smoke had developed arthritis and were indistinguishable from the controls, and this pattern remained until the end of the experiment. All mice, including controls, experienced a decrease in body weight during the development of CIA, but mice exposed to cigarette smoke had less weight loss during the acute phase of the disease and gained weight faster in the recovery phase (*P *< 0.05; Figure 1b). Seven weeks after collagen immunization, paws were examined histologically for synovitis and erosivity. At that time, mice exposed to cigarette smoke and controls had the same level of clinical arthritis and the same level of synovitis by histologic evaluation. Also joint destruction did not exhibit significant changes; however, animals exposed to cigarette smoke had been only affected in 37% of cases as compared with over 60% in the control group which indicates a less severe systemic disease (Figure 1c). Serum IL-6 in mice exposed to cigarette smoke and controls at the time of discontinuation (seven weeks after first immunization) was similar indicating that IL-6 is not significantly affected by cigarette smoke exposure (Figure 1d).

### Effect of nicotine on collagen induced arthritis

**Figure 2:**
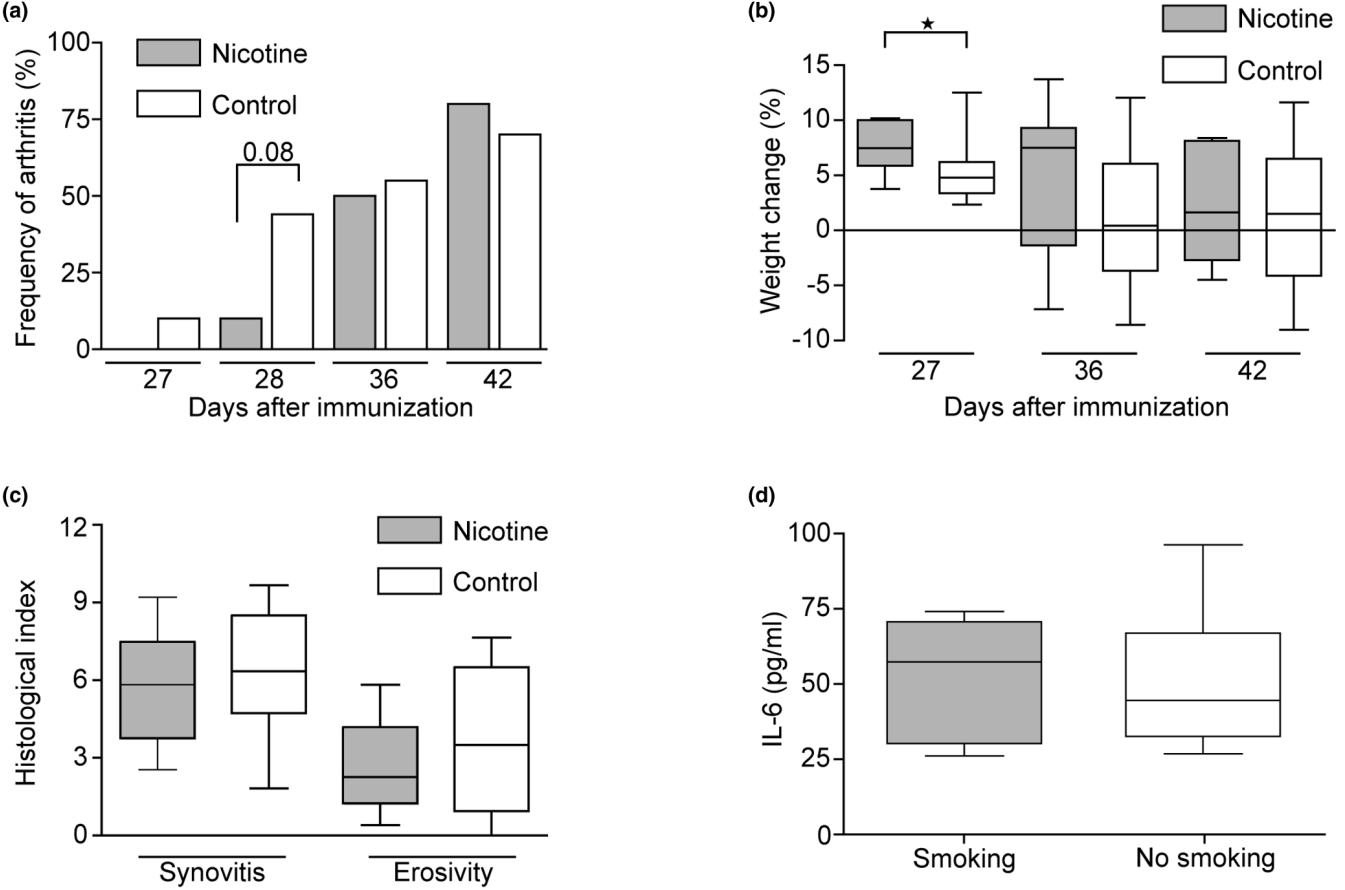
Delayed development and progression of collagen-induced arthritis in DBA/1 mice supplied with 0.01% nicotine in water. **(a) **Clinical signs of arthritis were followed between days 21 and 46 after immunization. Statistical evaluation was performed using chi-squared test. Development of arthritis was significantly delayed in smoking animals (*P *< 0.05). **(b) **Weight development after immunization with collagen II. Weight change was calculated as the change in percent as compared with the day of booster injection with collagen II. **(c) **Histologic changes in the joints were evaluated at day 46. **(d) **Levels of IL-6 were measured in serum at day 46 after first immunization. **(b to d) **Statistical evaluation was made using Mann-Whitney U test. Values are presented as mean ± standared error of the mean. Horizontal lines indicate medians. Nicotine treated group and control group contained 10 animals each.

In the second set of experiments, nicotine (100 μg/ml, concentration 0.01%) was provided in the drinking water during the entire course of the experiment. The amount of nicotine in water ingested per mouse was calculated to be 3 ml/day, which gives a total intake of 300 μg nicotine per day per mouse. Mice supplemented with nicotine did not show any significant reduction in the frequency of arthritis (Figure 2a), although a clear tendency for delayed onset can be seen at day 27 to 28 after immunization with only 10% of treated animals exhibiting signs of arthritis as compared with 40% in the control group (*P *= 0.08, not significant). Similar to the experiments with cigarette smoke exposure, nicotine-treated animals showed less weight reduction during the first phase of CIA as compared with the control group (*P *< 0.05; Figure 2b), suggesting that nicotine had a protective effect against the onset of arthritis. At day 36 after immunization, the frequency of arthritis is equally high in the nicotine-treated group as in the control group (50%). Histologic evaluation showed no significant effect of nicotine treatment either on presence of synovitis or erosivity, with mean arthritis index being on the same level in both groups (Figure 2c). Also, IL-6 levels measured at the time of discontinuation did not showed statistically significant differences as compared with the control group (Figure 2d).

### Ameliorating effect of cigarette smoke exposure on antibody production

**Figure 3:**
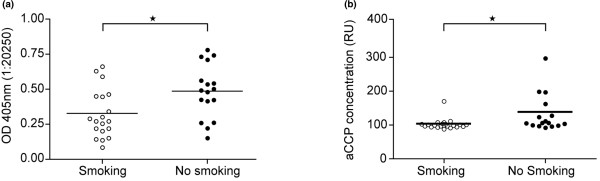
Reduction of serum type II collagen-specific and aCCP antibody levels after prolonged cigarette smoke exposure in mice with collagen-induced arthritis. Serum levels of autoantibodies to **(a) **collagen II and **(b) **anti-cyclic citrullinated peptides (aCCP) antibodies (n = 37), were measured on day 46 after first immunization using ELISA. Sera from two independent experiments were used. Data are presented as dot plots showing median and interquartile range. Mann-Whitney U test was used for statistical analysis.

Taking into consideration the role of antibody production in the pathophysiology of CIA, to determine whether cigarette smoke exposure influences their presence and levels, we measured levels of specific antibodies to collagen II as well as levels of anti-CCP antibodies in mice sera at the time of discontinuation of the experiment (days 42 to 46 from first immunization). Levels of specific IgG against collagen II in mice exposed to cigarette smoke were significantly lower seven weeks after immunization compared with non-smoking controls (*P *< 0.05) in two independent experiments (Figure 3a). However, animals treated with nicotine did not show statistical differences in levels of specific antibodies to collagen type II (data not shown). These data are consistent with clinical findings showing no differences in arthritis index between the groups (Figure 2a). Intriguingly, the number of aCCP-positive animals was significantly lower in mice exposed to cigarette smoke, with only one out of 22 animals testing positive for IgG aCCP whereas in the non-smoking controls five out of 16 were positive (*P *< 0.05; Figure 3b). The total frequency of aCCP in CIA was low (5/38 mice, 13%). These data clearly indicate that cigarette smoke decreased the ability to produce antibodies against collagen II and aCCP.

### Nicotine suppresses production of IL-6 by splenocytes

**Figure 4:**
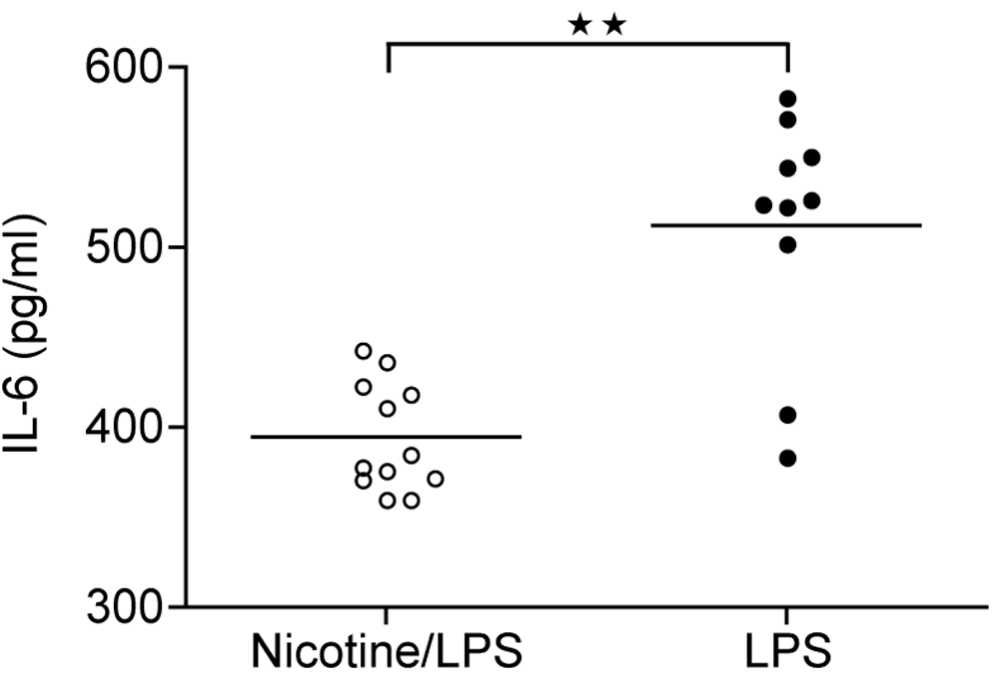
Nicotine decreases production of IL-6 by spleen cells *in vitro*. Levels of IL-6 in supernatants of naive spleen cells from NMRI mice after incubation for 24 hours with lipopolysaccharide (LPS) (1 μg/ml) in the presence or absence of nicotine (10 μg/ml) was measured by bioassay. Nicotine was added to cultures prior to LPS stimulation. Data are representative from three independent experiments (n = 3). Data are presented as dot plots showing median and interquartile range. Mann-Whitney U test was used for statistical analysis. Horizontal lines indicates medians.

To investigate a role of nicotine on the production of pro-inflammatory cytokines, LPS (10 μg/ml) was used to stimulate native NMRI splenocytes. As shown in Figure 4, cells incubated in the presence of nicotine with LPS after 24 hours produced a significantly lower amount of IL-6 (*P *< 0.005) as compared with controls. Data are representative of two independent experiments (n = 4). Levels of TNFα measured in the supernatants after 24-hours stimulation did not differed significantly (data not shown) in between the groups.

## Discussion

RA is an autoimmune disease manifested by severe joint inflammation, which leads to joint destruction. These processes are mediated by both innate and acquired immune systems. Cigarette smoking is currently considered a risk factor for the development of RA, and a factor in the progression of joint damage, although the underlying mechanisms remain unknown. Based on recent findings it has been postulated that there is a link between smoking and modification of the shared epitope alleles of the HLA-DRB1 gene, which is an acknowledged genetic risk factor for RA. It was also suggested that a combination of shared epitopes and smoking would increase the risk of developing RA [26]. However, to our knowledge, no experimental work has been carried out to validate the role of susceptibility genes and smoking on the development of autoimmune arthritis in mice. Extensive studies on transgenic mice expressing human susceptibility genes show that HLA-DQ and HLA-DR might be involved in susceptibility to or protection from CIA [27,28]. Studies with transgenic CIA mice and smoking have not been performed. Here we demonstrate that prolonged exposure to cigarette smoke has no negative impact on CIA but may even delay the onset and progression of arthritis in this model. Systemic inflammation was less severe in mice exposed to cigarette smoke, indicated as a milder decrease in weight occurred during the acute phase of the disease as well as a faster weight gain in the recovery phase.

How does then prolonged exposure to cigarette smoke delay onset of arthritis? A first line of evidence in support of a direct influence of smoking on the development of CIA comes from the collagen II antibody levels. At the end of the experiment the mice exposed to cigarette smoke showed strikingly lower levels of collagen II antibodies than non-smoking controls. Analogously, mice exposed to cigarette smoke developed significantly lower levels of antibodies to citrullinated peptides. Although the pathogenic role of aCCP in CIA is uncertain, we believe that cigarette smoke may reduce autoimmune responses in mice. The frequency of aCCP in our CIA model was low: only 13% had detectable aCCP, which is in agreement with previous reports [7,8] The possibility that smoking affects antibody production comes from human studies [29,30]. Thus, the frequency of response to vaccine is lower in smokers compared with non-smokers [29], and the reduction in serum Ig is not related to a decreased number of B cells but rather with suppression of their function and IgG. These effects of smoking are fully reversible [30]. The data suggest a potential immunosuppressive effect of cigarette smoke on animals and could explain the decreased levels of specific IgG in sera.

How do mice exposed to cigarette smoke correspond to smoking in humans? The median life span of a DBA/1 mouse is about two years (104 weeks). The mice were subjected to CS for a total of 22 weeks which equals about one-fifth of their lifespan (22/104). This will correspond to 16.9 years of smoking in humans with a median life span of 80 years. Indeed, chronic lung inflammation and destruction of alveolar walls induced by smoke exposure in mice show major similarities to the histologic changes observed in tobacco users [22]. Although we are so far unable to clearly identify components of cigarette smoke responsible for delaying the onset of disease, we believe that nicotine is at least partially involved. It is plausible that nicotine might play a role because it has been shown to have potent anti-inflammatory effects in experimental ulcerative colitis [13], improve outcome of sepsis through its effect on inflammatory mediators [14], and block leukocyte recruitment [15]. IL-6 is believed to be responsible for local and systemic inflammation processes in RA. This cytokine is required for the induction of CIA as mice with targeted inactivation of the IL-6 gene are resistant to CIA. In our *in vitro *experiments with LPS-stimulated spleen cells from naive mice, we observed that nicotine resulted in a striking decrease in IL-6 levels in supernatants compared with controls. However, levels of TNFα were unaffected when measured after 24 hours of incubation, which we believe is due to the fact that production and release of TNFα following LPS stimulation peaks as early as one hour after induction [31]. At the time of discontinuation of the experiment (days 42 to 46) frequency of arthritis was equalized and histologic evaluation of arthritis did not present any statistically significant changes, although we can clearly see the trend in number of affected animals.

No significant differences in IL-6 levels and TNFα in mice sera were measured. We believe that once inflammatory process is fully triggered, the modulating effect of cigarette smoke is too weak to significantly influence the outcome. Based on this evidence we propose nicotine as one of the molecules responsible for significant delay in onset of disease in animals exposed to smoke by decreasing inflammatory response to stimuli.

Recent studies provided some insights into the role of nicotine recognition in regulation of inflammation. Van Maanen and colleagues has recently shown that the use of nicotine and an agonist of the nicotinic acetylcholine receptor subunit α7 reduced severity of clinical signs of arthritis in a CIA model as well as TNFα expression in the murine synovial tissue [32]. In the human setting, the expression of the α7 subunit of the nicotinic receptor in synovia is inversely related to the local production of pro-inflammatory cytokines [33].

Another way in which nicotine could be responsible for reduced inflammatory response in our experimental model could be via expression of a glutamate receptor [34]. Glutamate levels are significantly increased in synovial fluids from patients with arthritis [18] and play a pivotal role in the development of edema and synovitis [35]. We suggest that nicotine present in cigarette smoke might bind to glutamate receptors (NMDA and mGlu) and limit its availability to bind glutamate as well as downregulate glutamate receptor expression in synovial tissue. The possible ameliorating effect of nicotine on arthritis mediated through modulation of the autonomous nervous system cannot be ruled out. As nicotine stimulates nicotinic acetylcholine receptors in both the parasympathetic and sympathetic division (through the splanchnic nerve to the adrenal medulla causing release of epinephrine) the total effect on arthritis via this way cannot be identified. It has been shown that the sympathetic nervous system supports inflammation during the asymptomatic phase of CIA, whereas it inhibits inflammation during the chronic symptomatic phase [36].

## Conclusions

Contrary to results from epidemiologic studies, we show in this study that smoking delays onset and slows down the progression of destructive arthritis in mice with CIA. The key substance in cigarette smoke, nicotine, may play an important role by alleviating inflammatory responses.

Although the results of the present study suggest that smoking or nicotine may not be detrimental to individuals at risk for RA or those who already have RA, our results should not be interpreted as support for the safety of smoking among individuals with RA. It is of interest, however, that these results differ greatly from those of several epidemiologic studies indicating an increased risk of developing RA when smoking [37-39].

## Abbreviations

aCCP: anti-cyclic citrullinated peptides; CIA: collagen-induced arthritis; ELISA: enzyme-linked immunosorbent assay; FCS: fetal calf serum; H&E: hematoxylin and eosin; IL: interleukin; LPS: lipopolysaccharide; RA: rheumatoid arthritis; TNFα: tumor necrosis factor alpha.

## Competing interests

The authors declare that they have no competing interests.

## Authors' contributions

SSL, PM, and I-MJ was responsible for the majority of the practical work and the writing of the manuscript. The study was originally designed by AT and MB. All authors were involved in different methodologic parts, the interpretation of data and writing of the manuscript. All authors read and approved the final manuscript.
